# Assessing the Impacts of Individual and Organizational Factors on South Korea Hotels’ Green Performance Using the AMO Model

**DOI:** 10.3390/ijerph191610440

**Published:** 2022-08-22

**Authors:** Taeuk Kim

**Affiliations:** Department of Hotel & Restaurant Management, Kyonggi University, Seoul 03746, Korea; teokim1305@naver.com

**Keywords:** green HRM practice, green awareness, green psychological climate, green organizational citizenship behavior, green performance management, green CSR perception

## Abstract

The goal of this study is to investigate the influence relationship between AMO applied green HRM and employees’ green OCB. This study also attempted to verify the relationship between employees’ green OCB and green performance management. Specifically, we tried to define the effects of green awareness and green psychological climate on green HRM and green OCB. Moreover, we examined the mediating effect of green CSR perception on the relationship between green OCB and green performance management. To do so, we surveyed Rooms and F&B employees from nine global chain hotels in South Korea that actively contribute to a green package. An “actively contributing hotel” is one that has leadership in energy and environmental design certification. Further, employees of such hotels consider them proenvironmental hotels. For data analysis, we carried out SEM and factor analysis using SPSS 18.0 and Amos 20.0 (IBM, Armonk, NY, USA). All hypotheses were adopted as having significantly positive (+) effects. Hypotheses 4, 7, and 11 predicted partial mediating effects. The findings of the study have significant theoretical and practical ramifications for corporate environmental strategic performance management. Specifically, this study identified the relationship between the mediation variables on green OCB and green PM, as demonstrated in previous studies. Additionally, these results provide an effective employee management strategy for the green HRM of green hotels by providing concrete data. First, green hotels need to raise employees’ green awareness and green psychological climate to increase their green OCB. Second, employees themselves ultimately have to enhance the green CSR perception to raise green PM.

## 1. Introduction

Environmental protection is a global issue [1]. Increasing discourses about environmental issues and consumers’ interest in protecting the environment has provoked companies to consider environmental discussions as essential. Moreover, contributing to the environment is now considered a crucial factor of corporate social responsibility (CSR) that could affect a company’s reputation and achievements [2,3]. Thus, corporations are meeting this social need through proenvironmental management and by making sustainable profits [4]. While the hotel industry is showing high energy consumption and waste production (e.g., disposables), hotels are attempting to become consumption-oriented businesses. This is evidenced by discussions about green hotels, which have emerged in the discursive field and have settled there [5]. The Sheraton Grand Incheon Hotel in South Korea acquired leadership in energy and environmental design (LEED) certification in the USA. It was designed to minimize the damage to nature caused by the structure’s design, construction, and operation. Likewise, the Grand Hyatt Incheon Hotel earned the gold grade from LEED by the U.S. Green Building Council (USGBC) by adopting air conditioners, refrigerators, and refrigerants with low potential to destroy the ozone layer or cause global warming. However, previous studies about business performance have focused on consumers. Further, the hotel sector depends heavily on human resources [6]. As HR management is important in proenvironmental management [7], this study will take the proenvironmental angle and determine whether the person–organization outcome can affect business performance.

This study intends to provide a better comprehension of organizations’ environmental goals and green HRM policies while also identifying possible changes in employees’ environmental recognition and sense of responsibility [8]. This can induce a proenvironmental attitude in employees and prevent undesirable incidents [9]. This situation, in turn, can encourage environmentally responsible behavior [10]. Employees deliver a company’s proenvironmental value through products or services. We tried to determine the effect of proenvironmental management on HRM, and proenvironmental organizational performance [11,12,13,14,15,16] and business performance [17,18,19,20,21].

Several variables have been studied in this regard. First is the influence variables suggested as result variables [15,22,23,24]. Second is the effectiveness of using employees’ quality and workability as the core of business performance. Third, and last, is organizational citizenship behavior and its traits as strong predictors of business performance. However, there are also considerable counterexamples, indicting the negative influence of employees’ capabilities on business performance. In this context, corporate management performance related to the environment stem from the actions of employees through human resource management, which aims for environmental management or performance, and these employee behaviors were verified in a study by [12,15,25] as a variable of green organizational citizenship behavior. Green OCB can be defined as personal and discretionary social behavior that is not expressly acknowledged by the official remuneration system and contributes to the organization’s more effective environmental management [26]. Thus, we tried to define the mediating effect of corporate green CSR perceived by employees. Specifically, we attempted to confirm the mediating impact of green CSR perception in the connection between employees’ green organizational citizenship behavior (OCB) and green performance management.

Moreover, we tried to confirm the effect of individual green OCB and green HRM at the organizational level. HRM is defined as education and training to develop employees’ talents and capabilities to enhance business performance [7]. Thus, we explored the influence relationship between employees’ green OCB and green HRM for employees’ education and training. Additionally, we applied the ability–motivation–opportunity (AMO) model to green HRM to improve our predictions of employees’ behavior. HRM can establish employees’ abilities by motivating them with high salaries and incentives and giving them opportunities to show their abilities by offering proposal systems or company contests. Such incentives affect employees’ and corporations’ performances [27].

We also tried to verify the roles of green awareness and green psychological climate in the development of employees’ green OCB. We expected to observe a meaningful influence relationship between variables and green performance. To sum up, based on existing studies [15,28,29,30] that have attempted to predict employees’ behavior based on green human resource management applied with the AMO model in the hotel industry, the mediating function of green psychological climate was additionally confirmed. In addition, the importance of CSR, mentioned above, is differentiated from existing studies by confirming the mediating function of CSR perceived by employees, not at the customer level, by verifying the influence relationship between variables from two dimensions. The first is the individual view of the OCB from global hotel employees in Korea; the second is the organizational view of green business performance. With these, we tried to confirm the influence relationship between HRM practice and green OCB. We also tried to verify the influence relationship between green awareness and psychological green climate/individual green OCB and organizational green performance management. Lastly, we assessed the mediating effect of green CSR perception.

## 2. Theoretical Background

### 2.1. Green Human Resource Management Applying the Ability–Motivation–Opportunity Model in Pro-Environmental Hotels

The proenvironmental approach is no longer limited to selling related products to consumers. Now, it is also important to reflect a corporation’s proenvironmental value on its delivery, the employees. Such efforts are gaining attention from many related individuals and entities [11,12,13,14,15,16].

Thus, corporations need to reflect on how proenvironmental values are implemented in every process that they carry out, including recruiting, training, promoting, rewarding, managing, and directing. It is considered that with these, a corporation’s proenvironmental management can possess longevity. According to recent studies, corporations that engage in proenvironmental management are more appealing to fine human resources [31]. This structure can be improved by applying a system that rewards employees’ proenvironmental behavior [32]. It has also been shown that a manager’s initiative for proenvironmental values can also promote changes in employees [33].

A question arises from this context: Can a company enhance its competitiveness and performance by launching such a human resource management system? This question has been one of the most important research subjects in HR management studies. We attempted to answer this question by establishing an AMO model based on many studies and theoretical approaches. The AMO model conceptualizes the factors that can enhance an organization’s competitiveness and performance—namely, ability, motivation, and opportunity [34,35]. In other words, an HR management system can build human resources’ abilities, motivate them with high salaries and incentives, and give them opportunities by offering a proposal system or company contest to affect both individual/organizational performance [27].

The positive relationship between HR systems and various organizational performances has received much empirical support. However, it is not clear how an HR system can affect both individual and organizational performance [36]. Becker and Gerhart [37] stressed the importance of finding a new variable that acts as the bridge in an HR system’s value creation. Reverse causality issues arise when the mediated performance or employees’ individual awareness/organizational climate is not measured. Thus, the understanding of the influence relationship between business performance and HR systems can be halted. Various studies have been conducted in this context. Researchers have tried to find the bridge variable between HR management and organizational performance by realizing how HR management systems improve business performance, and the number of such studies continues to increase [38].

### 2.2. The Link between Green Human Resource Management, Green Awareness, Green Psychological Climate, and Green Organizational Citizenship Behavior

Snell et al. [39] defined strategic HR management as “types of planned human resource deployment and activities to achieve organizational goals.” Meanwhile, “strategic management” refers to all organizational activities related to organizational performance. In this context, the primary focus of strategic HR management is to define the mechanisms connecting a company’s HR system to its organizational performance.

HRM should motivate employees to join organizational environment management. Environmental awareness is a multifaceted concept known as an influencer that can affect individual attitudes, information, knowledge, tendencies, behaviors, intentions, efforts, and activities [40]. Additionally, it is connected to psychological elements that influence people’s attitudes and activities, as well as whether they engage in proenvironmental behavior [41]. High recognition of environmental issues helps personnel who should be made aware of the value of environmental conservation for their well-being [42]. It also helps employees recognize nature’s vulnerability and the importance of keeping it safe. Improvements to environmental awareness requires a thorough knowledge of environmental issues. This is a practical method for simultaneously enhancing environmental performance and proenvironmental behavior. Thus, environmental awareness is an important factor in proenvironmental management [11].

Forming a favorable organizational climate is also important. Changing employees’ working environment and improving their business participation and focus are related to their business awareness [43]. Researchers have verified that the business participation of employees improves if the organization has a positive climate [44,45,46,47]. Regarding the efficacy of HR teams, many researchers have focused on how HR teams can promote HR development. For instance, they can measure the education or behavioral correction done following the organization’s main objective [48]. HRM practice can raise employees’ job satisfaction and organizational involvement. As a result, employees can improve their morale and awareness of doing the right thing, which HRM practice aims to achieve [49]. Therefore, green HRM raises employees’ green awareness for environmental protection [50], which raises green OCB [51,52]. A meaningful influence relationship between environmental HRM and employees’ psychological climate has been verified as well [53,54,55].

Various studies have verified the influence relationship between the AMO-applied HRM and employees’ attitudes and behaviors. For instance, Pham et al. [23] showed the effect of applying the AMO model to HRM practice on employees’ environmental commitment, environmental OCB, and corporate environmental performance in three to five star hotels in Vietnam. Pham et al. [46] also confirmed the influence relationship between green rewards, green performance, and environmental OCB. López-Gamero et al. [47] found an influence relationship between HRM based on the AMO model and environmental management, while also showing positive effects of environmental management on cost-competitive advantage and differentiation–competitive advantage, as well as positive effects of RevPAR, average daily rate, and the inefficacy of occupancy on performance. Aboramadan and Karatepe [56] verified that environmental HRM has a meaningful impact on green organizational citizenship behavior, and we based our hypothesis on their research.

**Hypothesis** **1** **(H1).** *The image perception of GCSR has a significant effect on GC.*

**Hypothesis** **2** **(H2).** *GPB has a significant effect on GC.*

**Hypothesis** **3** **(H3).** *GA has a significant effect on GOCB*.

**Hypothesis** **4** **(H4).** *GA has a significant mediating effect on the relationship between GHRM and GOCB*.

**Hypothesis** **5** **(H5).** *Green HRM has a significant effect on PGC*.

**Hypothesis** **6** **(H6).** *PGC has a significant effect on GOCB*.

**Hypothesis** **7** **(H7).** *PGC has a significant mediating effect on the relationship between GHRM and GOCB*.

### 2.3. The Link between Green Corporate Social Responsibility Perception, Green Organizational Citizenship Behavior, and Green Corporate Performance Management

CSR has gained much attention in the field of sustainability and the environment. According to [57], CSR can indicate a corporation’s devotion to individuals who can be affected by society or social activity. CSR is also related to organizational devotion to human society, to which the goals and intentions of an organization are imperative. Without those, the beneficial use of CSR to the interested parties and businesses (e.g., ethical or economic support for welfare or culture) cannot be done flawlessly [58]. Promoting CSR regulations for efficient environmental protection can advance the ethics related to social and corporate responsibility, which can directly resolve the social [59]. Abbas et al. [60] showed that corporations with social responsibility take on concerned persons and customers thereby have a positive image of these companies. The importance of CSR is also indicated by the relationship between the persons concerned and corporations.

In this context, Lee [61] concluded that CSR can be a successful strategy by which a corporation can gain higher recognition from the public and persons concerned. Ismail [62] claimed that a portion of the world’s biggest enterprises made tangible pledges of CSR by minimizing environmental impacts to grow their enterprise, improve social reliability, and secure their financial and environmental performance.

According to Roy et al. [63], establishing environmental management programs and forming organizational environmental policies depend on green OCB. Green OCB can interact with businesses’ performance and CSR to form even greater relationships. Employees have a role to improve the hotel’s green management performance [64], and green organizational citizenship behavior is a concrete form of proenvironmental behavior [65] which refers to the willingness of employees to cooperate with the organization and organizational members to enhance behavior beyond their job roles that benefit the natural environment without harming it.

In this context, Zhao and Zhou [66] showed the influence relationship between OCBE and green corporate performance management. This was possible because OCBE can induce proenvironmental goal settings and standards in corporate operations. Jiang et al. [67] and Valencia et al. [68] claimed that they contribute to improving corporate environmental goals and the daily life of normal people. Yeo [69] supported the idea that OCBE contributes to corporations’ proenvironmental performance management by improving business performance related to their socially responsible business behavior. Cho et al. [70] demonstrated a favorable impact of CSR activity on financial and environmental performance.

Studies have also verified that green CSR can only partly mediate the influence relationship between green transformational leadership and green performance [71]. Moreover, green CSR can partially mediate the influence relationship between green shared vision and green corporate performance [72]. The hypothesis of the present study was based on the theories stated above.

**Hypothesis** **8** **(H8).** *GOCB has a significant effect on GCSR*.

**Hypothesis** **9** **(H9).** *GCSR has a significant effect on GPM.*

**Hypothesis** **10** **(H10).** *GOCB has a significant effect on GPM*.

**Hypothesis** **11** **(H11).** *Green CSR perception has the mediation effect on the relationship between GOCB and GPM*.

## 3. Method

### 3.1. Measures and Questionnaire Development

**Figure 1:**
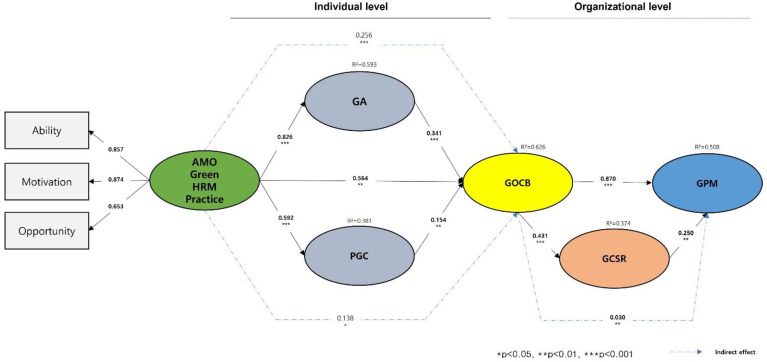
Structural equation model estimation and test for structural metric invariance. Note 1. Green HRM practice (GHRM), green awareness (GA), green psychological climate (GPC), green organizational citizenship behavior (GOCB), green performance management (GPM), green CSR perception (GCSR). Note 2. Goodness-of-fit statistics for the measurement model: χ^2^ = 802.227, df = 694, *p* < 0.001, χ^2^/df = 0.865, RMSEA = 0.022, CFI = 0.985, IFI = 0.985, TLI = 0.982, * *p* < 0.05, ** *p* < 0.01, *** *p* < 0.001. Note 3. Goodness-of-fit statistics for the measurement model: χ^2^ = 794.213, df = 691, *p* < 0.001, χ^2^/df = 0.870, RMSEA = 0.022, CFI = 0.986, IFI = 0.986, TLI = 0.983, * *p* < 0.05, ** *p* < 0.01, *** *p* < 0.001; Note 4. All factors’ loadings are significant at *p* < 0.001.

Two components made up the online survey that was used in this investigation. The survey’s initial section asked for demographic data (e.g., gender, age, marital status, educational level, position). The second part of the conceptual model included questions related to the constructs of the green HRM practice (GHRM), green awareness (GA), green psychological climate (GPC), green organizational citizenship behavior (GOCB), green performance management (GPM), and green CSR perception (GCSR). We assessed the study constructs by employing measures used in the existing literature [73,74,75,76]. A five-point Likert scale with the questionnaire items was used to evaluate the constructs. In particular, three subfactors (ability, motivation, opportunity) were assessed. Thirteen items adopted from [73] were used to assess green HRM. Four items adopted from [24], based on [77], were used to assess green awareness. Three items adopted from [74], based on [78], were used to assess the green psychological climate. Ten items adopted from [75], based on [79], were used to assess organizational citizenship behavior. Four items adopted from [76], based on [80], were used to assess green CSR perception. Seven items adopted from [81], based on [82,83], were used to assess green performance management. There is Figure 1. shows the relationship between the above variables.

A pretest with four Ph.D. students specializing in hotel management and five hotel employees with ten years of experience in the industry helped to develop this questionnaire. Through the process of modification and complementing, the questionnaire was finalized after a review by a human resource manager and professor in hotel management. In the Appendix A, every item used in this study is listed.

Procedures were corrected before research was conducted to reduce common method bias (CMB). Moreover, in order to protect responder privacy and ease evaluation anxiety, the questionnaire was created, thus reducing CMB [84,85]. Starting on 4 April 2022, we conducted a survey among Rooms and F&B employees from global chain hotels in South Korea for 25 days. Hotels were chosen based on their sincerity in following a green package. The HR managers of the hotels assisted in the distribution of the questionnaires. We administered 450 online and offline surveys and collected 379 responses after filtering out insincere answers.

### 3.2. Data Collection Process

To realize the empirical analysis, we used convenience sampling and judgment sampling (nonprobability) for our survey to verify the relationships between green HRM practice, green awareness, psychological green climate, green organizational citizenship behavior, green performance management, and green CSR perception. The subjects were green hotel employees in South Korea.

Before beginning the survey, we selected nine global chain hotels with LEED certification that had shown their employees’ dedication to a green package and CSR image recognition. The package includes ‘Earth Save Green’, ‘Sustainable Journeys’, ‘Green Stay’, ‘Spring Greenity’, which entails recycling waste linen, ‘Zero Waste Stay,’ which entails reduced waste, and ‘Amenity’ recycles waste linen. Adopted hotels with active green packages are likely to have proenvironmental policies, high customer satisfaction, and sufficient employee training on environmental performance.

By providing respondents with brief instructions about green HRM practices, we excluded participants who gave a response below 4 on the Likert scale for items regarding comprehension and recognition. To ensure that answers were sincere, we deployed drink vouchers to respondents.

### 3.3. Data Analysis and the Sample

We employed SPSS 20 and AMOS 20 to examine the data. As recommended by [86], before evaluating the proposed structural model, the measurement model with a Confirmatory Factor Analysis (CFA) was initially tested. The reliability and validity of the measuring items for each construct were assessed. The next step used a structural equation modeling (SEM).

Regarding the demographic characteristics of the 379 participants, 52.4% (*n* = 198) were female employees and 47.7% (*n* = 181) were male employees. Moreover, while 56.9% were unmarried (*n* = 216), 43.1% were married (*n* = 163). In addition, 33.7% (*n* = 128) of respondents were 30–40 years old, 30.6% (*n* = 116) of respondents were 40–49 years old, 16.6% (*n* = 63) of respondents were 20–29 years old, 14.5% (*n* = 55) of respondents were 50–59 years old, and 4.5% (*n* = 17) of respondents were 60 years old and above. Their levels of education were high school graduate and below (9.8%, *n* = 37), 2-year college (31.66%, *n* = 120), 4-year college (39%, *n* = 148), and postgraduate or higher (19.5%, *n* = 74). The positions were 40.7% (*n* = 167) agent, 23.9% (*n* = 98) supervisor, 17.3% (*n* = 71) assistant manager, 10.73% (*n* = 44) manager, and 7.3% (*n* = 30) director or above in order.

## 4. Results

### 4.1. Common Method Bias

The purpose of this study was to reduce Common Method Variance (CMV) by designing the proper survey and questionnaire. Two procedural remedies and analysis methods were used for embodiment. First, we used different cover stories for each respondent. Criterion variables and predictors were psychologically separated by doing so, while the applied scale was the same for all. An example of the cover story is instructed below; “The following statements are irrelevant to the above questions. Please carefully read each statement and then mark from extremely disagree to agree with your recent feeling”. Second, we focused on item ambiguity. As suggested by [87], for the purpose of improving respondents’ comprehension, we offered clear descriptions of ambiguous terminology. Moreover, unmeasured latent methods factor in CMV at 1.1 percent, according to the findings of Harman’s one-factor analysis, a post hoc test to identify potential CMV. It was determined that the data suited another one-factor measurement model well (goodness-of-fit statistics for the measurement model: χ^2^ = 655.238, df = 541, *p* < 0.001, χ^2^/df = 0.825, RMSEA = 0.027, CFI = 0.941, IFI = 0.943, TLI = 0.923). This study used procedural corrections during the survey design phase to reduce CMV. Therefore, CMV did not influence the parameter estimations.

### 4.2. Confirmatory Factor Analysis

To confirm the reliability and validity, confirmatory factor analysis (CFA) was used. The measurement model as a result, goodness-of-fit statistics for the measurement model χ^2^ = 794.213, df = 691, *p* < 0.001, χ^2^/df = 0.870, RMSEA = 0.022, CFI = 0.986, IFI = 0.986, TLI = 0.983, were judged to be overall excellent [88]. To examine the convergent validity of the measurement model’s latent variables, factor loadings, the significant probability of the t-value, the average variance extracted (AVE), and the construct reliability (CR) were examined. The confidence coefficients (Cronbach’s alpha) of the factor loading ranged from 0.599 to 0.917, which was significantly higher than the 0.6 indicated by [89]. Moreover, AVE values and CR values were respectively constructed ranging from 0.623 to 0.966 and from 0.868 to 0.988. These values were all higher than the ranges of 0.5 and 0.7 that were suggested by [86].

Additionally, correlation analysis was carried out, as shown in Table 1, to confirm the discriminant validity. As a result of Pearson’s correlation analysis, all variables of green HRM practice (GHRM), green awareness (GA), green psychological climate (GPC), green organizational citizenship behavior (GOCB), green performance management (GPM), and green CSR perception (GCSR) were *p* < 0.05, indicating a significant correlation association [90]. As a result, discriminant validity was verified.

### 4.3. Structural Model and Hypothesis Testing

In this study, by applying AMO, the first verification of the green HRM practice and the green OCB of hotel employees is conducted, and the second verification of green performance management is conducted accordingly. The maximum likelihood estimation method, which was used as an estimation method for both the model and procedure’s evaluation, was used to create the structural equation model (SEM) analysis [88]. The goodness-of-fit of structural model (χ^2^ = 802.227, df = 694, *p* < 0.001, χ^2^/df = 0.865, RMSEA = 0.022, CFI = 0.985, IFI = 0.985, TLI = 0.982) was satisfactorily higher than the standard value.

Moreover, the SEM had shown a high prediction power for R^2^(GA) = 0.593, R^2^(PGC) = 0.381, R^2^(GOCB) = 0.626, R^2^(GPM) = 0.508, R^2^(GCSR) = 0.374, and t-values and standardized path coefficient were shown as the result in Table 2. The path estimates show that GHRM had a significantly positive effect on GOCB (β = 0.564, t = 2.691 **), GHRM had a significantly positive effect on GA (β = 0.826, t = 7.941 ***), and GA had a significantly positive effect on GOCB (β = 0.341, t = 3.780 ***). Thus, H1, H2, and H3 were supported. Additionally, GA had a partially significant mediation effect of the relationship between GHRM and GOCB. Thus, H4 was supported. The results of the analysis of the mediation effect are shown via several significant indirect effects in Table 2.

GHRM had a significantly positive effect on PGC (β = 0.592, t = 7.428 ***) and PGC had a significantly positive effect on GOCB (β = 0.154, t = 2.201 **). Thus, H5 and H6 were supported. Additionally, PGC had partially a significant mediation effect of the relationship between GHRM and GOCB. Thus, H7 was supported. The results of the analysis of the mediation effect are shown via several significant indirect effects in Table 2.

GOCB had a significantly positive effect on GCSR (β = 0.431, t = 4.142 ***), GCSR had a significantly positive effect on GPM (β = 0.250, t = 2.235 **), and GOCB had a significantly positive effect on GPM (β = 0.870, t = 8.606 ***). Thus, H8, H9, and H10 were supported. Additionally, GCSR had partially a significant mediation effect on the relationship between GOCB and GPM. Thus, H11 was supported. The results of the analysis of the mediation effect are shown via several significant indirect effects in Table 2.

## 5. Conclusions

### 5.1. Discussion and Implication

There is a global need for ESG management in the hotel sector to overcome external influences such as COVID-19. This study suggested a way for hotel enterprises to overcome incidents, improve HR management via green HRM practices, and check the influence of their efforts. Specifically, we applied the AMO model to the green HRM practice as a causal variable to verify employees’ green awareness, green psychological climate, and green OCB. We also demonstrated the influence correlations between green OCB, green CSR perception, and green performance management in organizational aspects. The verification object is a Korea-based global chain hotel with active green package practice. The research outcomes and implications are described below.

The first hypothesis outcomes for the individual level are as follows.

H1: GHRM has a significant effect on GOCB (supported). H2: GHRM has a significant effect on GA (supported). H3: GA has a significant effect on GOCB (supported). H4: GA has a partially significant mediating effect on the relationship between GHRM and GOCB (supported). H5: GHRM has a significant effect on PGC (supported). H6: PGC has a significant effect on GOCB (supported). H7: PGC has a partially significant mediating effect on the relationship between GHRM and GOCB (supported).

The second hypothesis outcomes for the organizational level are as follows.

H8: GOCB has a significant effect on GCSR (supported). H9: GCSR has a significant effect on GPM (supported). H10: GOCB has a significant effect on GPM (supported). H11: GCSR has a partially significant mediating effect on the relationship between GOCB and GPM (supported).

### 5.2. Theoretical Implications

In the hotel sector, HRM is a crucial factor, as employees need to interact with customers directly. As the need for ESG management increases, we applied the AMO model to green HRM practice and attempted to explain the relationships between several variables. This study extended previous studies on HRM based on the AMO model [15,46,91,92] by using additional variables, which are green awareness, green psychological climate, and green CSR perception. Furthermore, while the structural studies of green performance’s causal variables [15,22,23,24] assessed the organizational level only, we also simultaneously checked the individual level of green OCB. In addition, in a study related to green hotel, basic evidence data and academic areas in future research were developed to investigate the relationship between employee behavior and green management performance through verifying the mediating effect of green CSR perception as perceived by employees and not CSR from a customer perspective.

### 5.3. Managerial Implications

We found a meaningfully positive influence relation between green HRM and green OCB. We also verified the positive influence relation between green OCB and green performance management. From the above description, we suggest that hotel companies prepare long-term plans for their employees’ ability, motivation, and opportunities to improve environmental performance management.

In addition, we verified some effects (including mediation) of green awareness, green psychological climate, and green CSR perception in the structural relationship between green HRM, green OCB, and green performance management. Hotels should improve their green psychological climate and employees’ awareness by performing strategic management so that interactive communication with employees can be efficiently carried out. This is because it is capable of grasping the direction and vision of the difference more clearly between employees’ green awareness and green psychological climate. For instance, companies can run small green communities to raise employees’ green awareness and green psychological climate by sharing green conservation resources. Doing so will highlight the necessity of hotel employees and raise their morale.

Hotels should also utilize various marketing strategies (e.g., running a hotel website or SNS to promote employees’ participation). For instance, managers can implement a quiz about implementing green conservation and reward winners with a green product. This will motivate employees and form a proenvironmental atmosphere within the organization.

Moreover, through green HRM, it is necessary to create a surrounding environment, such as a performance-oriented management method, in which employees can perceive and immerse themselves in green awareness. For example, work satisfaction, education and training hours, and the proportion of employees who participated in training are selected and evaluated as employee-related indicators. By this, the level of awareness and commitment of employees in jobs related to green management performance can be increased.

In addition, to improve the hotel’s the green image by providing opportunities such as an idea contest for employees in the organization in the process of establishing strategies for green marketing, green awareness, and green psychological climate related to green management performance and green OCB can be positively promoted.

### 5.4. Study Limitations and Future Research

Few participants in their 20s and their 60s or above took part in this study when compared to those in their 30s, 40s, and 50s. Most of those in their 20s were agents or supervisors, the primary position of customer reception. In this context, this study is limited in its observations of millennials’ intentions and behaviors. Further research should be conducted with a more balanced age model.

The questionnaire used in this study was administered quantitatively to verify the structural relationships between variables. However, it can be hard to make a Likert scale for factors such as green awareness, psychological green climate, and green CSR perception, as they are psychological constructs. We suggest using a hybrid research method to achieve qualitative research and raise the sample’s representability.

We interviewed employees from Korean branches of a global chain hotel with active green package participation. However, hotels with inactive green packages can also have active HRM practices in an ESG management context and could already be improving their environmental performances. Therefore, future research should consider a better criterion for green hotels. A comparative study between local hotels and global chain hotels could be meaningful.

## Figures and Tables

**Table 1:** The measurement model and correlation.

Construct and Scale Item		Standardized Loading	Mean (SD)	AVE (CR)	GHRM	GA	PGC	GOCB	GPM	GCSR	√AVE
GHRM	GHRM1	0.917	3.62 (0.520)	0.966 (0.988)	1						0.983
	GHRM2	0.878									
	GHRM3	0.607									
GA	GA1	0.679	3.68 (0.609)	0.623 (0.868)	0.542 ***	1					0.789
	GA2	0.652									
	GA3	0.666									
	GA4	0.725									
	GA5										
PGC	PGC1	0.703	3.67 (0.612)	0.722 (0.886)	0.065 **	0.028 **	1				0.85
	PGC2	0.837									
	PGC3	0.723									
GOCB	GOCB1	0.587	3.98 (0.408)	0.674 (0.947)	0.958 ***	0.554 ***	0.075 *	1			0.821
	GOCB2	0.668									
	GOCB3	0.753									
	GOCB4	0.706									
	GOCB5	0.630									
	GOCB6	0.599									
	GOCB7	0.646									
	GOCB8	0.631									
	GOCB9	0.639									
	GOCB10	0.680									
GPM	GPM1	0.712	4.17 (0.420)	0.707 (0.944)	0.313 ***	0.280 ***	0.046 *	0.341 ***	1		0.841
	GPM2	0.674									
	GPM3	0.688									
	GPM4	0.704									
	GPM5	0.694									
	GPM6	0.562									
	GPM7	0.673									
GCSR	GCSR1	0.777	3.65 (0.460)	0.922 (0.979)	0.192 ***	0.244 ***	0.043 *	0.191 ***	0.242 ***	1	0.96
	GCSR2	0.791									
	GCSR3	0.888									
	GCSR4	0.782									

Note 1. SD = standardized deviation, AVE = average variance extracted, CR = composite reliability; green HRM practice (GHRM), green awareness (GA), green psychological climate (GPC), green organizational citizenship behavior (GOCB), green performance management (GPM), green CSR perception (GCSR). Note 2. Goodness-of-fit statistics for the measurement model: χ^2^ = 794.213, df = 691, *p* < 0.001, χ^2^/df = 0.870, RMSEA = 0.022, CFI = 0.986, IFI = 0.986, TLI = 0.983, * *p* < 0.05, ** *p* < 0.01, *** *p* < 0.001. Note 3. All factors’ loadings are significant at *p* < 0.001.

**Table 2:** Hypothesis testing.

Hypothesized Paths	Coefficients	t-Values
H1: GHRM → GOCB	0.564	2.691 **
H2: GHRM → GA	0.826	7.941 ***
H3: GA → GOCB	0.341	3.780 ***
H5: GHRM → PGC	0.592	7.428 ***
H6: PGC → GOCB	0.154	2.201 **
H8: GOCB → GCSR	0.431	4.142 ***
H9: GCSR → GPM	0.250	2.235 **
H10: GOCB → GPM	0.870	8.606 ***
H1: GHRM → GOCB	0.564	2.691 **
H2: GHRM → GA	0.826	7.941 ***
H3: GA → GOCB	0.341	3.780 ***
	Indirect effect	Total effect
H4: GHRM → GA → GOCB	0.265 ***	0.957 ***
H7: GHRM → PGC → GOCB	0.138 *	0.821 ***
H11: GOCB → GCSR → GPM	0.030 **	0.299 ***
Explained variable:	R^2^(GA) = 0.593, R^2^(PGC) = 0.381, R^2^(GOCB) = 0.626, R^2^(GPM) = 0.508, R^2^(GCSR) = 0.374	

Note 1. green HRM practice (GHRM), green awareness (GA), green psychological climate (GPC), green organizational citizenship behavior (GOCB), green performance management (GPM), green CSR perception (GCSR). Note 2. Goodness-of-fit statistics for the measurement model: χ^2^ = 802.227, df = 694, *p* < 0.001, χ^2^/df = 1.156, RMSEA = 0.022, CFI = 0.985, IFI = 0.985, TLI = 0.982, * *p* < 0.05, ** *p* < 0.01, *** *p* < 0.001. Note 3. All factors’ loadings are significant at *p* < 0.001.

## Data Availability

Data sharing is not applicable.

## Appendix A

**Green HRM**
Act
We develop training programs in environment management to increase environmental awareness, skills and expertise of employees We have integrated training to create the emotional involvement of employees in environment management We have green knowledge management (link environmental education and knowledge to behaviors to develop preventative solutions)
Motivation
We use green performance indicators in our performance management system and appraisals Our hotel sets green targets, goals, and responsibilities for managers and employees. In our hotel, managers are set objectives on achieving green outcomes included in appraisals In our hotel, there are disbenefits in the performance management system for noncompliance or not meeting environment management goals
Opportunity
Our hotel has a clear developmental vision to guide the employees’ actions in environment management In our hotel, there is a mutual learning climate among employees for green behavior and awareness in my company In our hotel, there are a number of formal or informal communication channels to spread green culture in our company In our hotel, employees are involved in quality improvement and problem-solving on green issues We offer practices for employees to participate in environment management, such as newsletters, suggestion schemes, problem-solving groups, low-carbon champions, and green action teams Our hotel emphasizes a culture of environmental protection
Green Awareness
The effects of pollution on public health are worse than we realize Over the next several decades, thousands of species will become extinct Claims that current levels of pollution are changing earth’s climate are exaggerated (reverse coded) Environmental protection will provide a better world for me and my children.
Psychological Green Climate
Our organization is interested in supporting the efforts made to handle environmental problems Our organization believes that it is important to protect the environment Our organization is concerned about working in a more environmentally friendly way
Green Organizational Citizenship Behavior
In my work, I weigh the consequences of my actions before doing something that could affect the environment I voluntarily carry out environmental actions and initiatives in my daily work activities I make suggestions to my colleagues about ways to protect the environment more effectively, even when it is not my direct responsibility I actively participate in environmental events organized in and/or by my hotel. I stay informed of my hotel’s environmental initiatives I undertake environmental actions that positively contribute to the image of my hotel. I volunteer for projects, endeavors, or events that address environmental issues in my hotel I spontaneously give my time to help customers take the environment into account every time they stay at this hotel I encourage customers to adopt more environmentally conscious consumption when they stay I encourage customers to share their ideas and opinions on environmental issues concerning this hotel
Environmental CSR
Our hotel implements special programs to minimize its negative impact on the natural environment Our hotel participates in activities which aim to protect and improve the quality of the natural environment Our hotel targets sustainable growth which considers future generations Our hotel makes investments to create a better life for future generations
Environmental Performance
Environmental management within our hotel has reduced waste Environmental management within our hotel has conserved water usage Environmental management within our hotel has conserved energy usage Environmental management within our hotel has reduced purchases of nonrenewable materials, chemicals, and components Environmental management within our hotel has reduced overall costs Environmental management within our hotel has improved its position in the marketplace Environmental management within our hotel has helped enhance the reputation of our hotel
